# 

**Published:** 1894-03

**Authors:** 


					﻿S yr	Y V 1	MOJVTfltY AT THREE DOLIAB8 A TEAR. rxr 7*
VOL. AV.J	SINGLE COPIE8, THIRTY CENTS.	LW'Oi 3*'
------------------------------------------ ------------- - ---------------Jf	Vip ffi
Entered at the Post Office, February 3rd, 1891, at New York, as Second-class Matter ’ * * do;
Copyright, 1893, by W. A. Conklin and R. 8. Huidekoper.	X	q
Printed for the Editors by E. Scott Co., 146 West 23d Street, New York.	.
THE JOURNAL
OF
COMPARATIVE MEDICINE
AND
VETERINARY ARCHIVES.
EDITED BY
W. A. CONKLIN, Ph. D., D. V. S.
RUSH SHIPPEN HUIDEKOPER, M. D., Veterinarian, (Alfort,)
ALETT YORK.
MARCH, 1894.
Ail communications and payments should be addressed to
MANAGING EDITOR JOURNAL COMP. MEDICINE AND VET. ARCH.
Station G-, New York City.
Copies may be had In London of BAILLIERE, TINDALL & COX.
				

